# Estimation of space-time self-exciting point process models using multi-dimensional Gaussian-type exponent approximation

**DOI:** 10.1371/journal.pone.0345865

**Published:** 2026-04-02

**Authors:** Edward Appau Nketiah, Chenlong Li, Weihua Yang, Yingchuan Jing, Ping Guo

**Affiliations:** College of Mathematics, Taiyuan University of Technology, Jinzhong, Shanxi, China; University of Houston, UNITED STATES OF AMERICA

## Abstract

Space-time self-exciting point process models are introduced to capture the clustering features in crime datasets. It does particularly well in modeling social network datasets, crime and security datasets, financial datasets, and seismic datasets. However, there has been limited analysis of large crime datasets using the space-time self-exciting point process models due to a lack of flexibility in the estimation of the conditional intensity function and computational challenges associated with large datasets. To explore the applicability of these models for crime data, we propose a multi-dimensional Gaussian-type exponent approximation method. This method addresses computational difficulties associated with large datasets and enables flexible estimation of the conditional intensity function. We evaluate the proposed method through simulations and apply it to study the space-time patterns of burglaries in Chicago, Illinois, United States. The results demonstrate that the proposed method is flexible, has overcome computational difficulties, and reveals a strong clustering phenomenon in the burglary data.

## 1 Introduction

Space-time point process models have gained significant attention as an extension of temporal point process models by incorporating spatial components. The space-time self-exciting point process model predicts the occurrence of events as a function of spatial components, time, and previous historical events; when an event happens, the likelihood of subsequent events increases. The traditional space-time Poisson process models are limited for analyzing this type of event because they assume conditional independence and do not account for history dependence, even when applied to inhomogeneous settings; hence, the space-time self-exciting point process models are introduced to handle such situations. According to [1], it is intuitively natural to specify a space-time self-exciting point process model by its conditional intensity function at a certain location and time, given the past events of the process up to that time.

In temporal self-exciting point process models, the occurrence of an event triggers other events to happen [2]; similarly, in space-time self-exciting point process models, the occurrence of events across spatial and temporal dimensions triggers other similar events to occur around or at the same location at a certain point in time. These models have been observed to capture clustering and triggering behaviors naturally, and have been applied in areas where spatiotemporal events occur, such as criminology [3,4], epidemiology [1], earthquakes [5], etc. The conditional intensity function of the self-exciting point process model is partitioned into two components: a background rate or intensity and the triggering effect. For instance, in seismology, the occurrence of a major earthquake triggers other minor earthquakes, called aftershocks, to occur. The main earthquake occurs independently as a result of the background intensity, while the aftershocks, triggered by the main earthquake, represent the triggering effect.

Due to the clustering of spatiotemporal events, it is well known that when an earthquake occurs, it increases the likelihood of subsequent earthquakes in nearby regions over a short period of time [5]. To model this behavior, the parametric Epidemic-Type Aftershock Sequence (ETAS) model was proposed for earthquake datasets [6]. In the case of criminology, the spread of crime results in the formation of crime clustering patterns in space and time, studied by [3]. A study into criminology has shown that crime is spatially concentrated or location-specific, and its continual occurrence follows a “contagion-like” process [7]. For instance, burglars may target a particular locality frequently due to its vulnerability [8]. The application of self-exciting point process models in criminology, specifically using the Hawkes process, was first studied by [3], with subsequent research expanding on the use of self-exciting process models [9,10].

Moving forward, [11] also investigated the spread of crime in a spatiotemporal setting and assessed the predictive performance of space-time self-exciting point process model for assault and burglary crimes. The use of basis function to investigate the triggering effect and background intensity have been studied [12,13]. [12] research focuses primarily on estimating the triggering function through iterative algorithm while [13] studies emphasize on combining maximum likelihood estimation (MLE) with group-lasso regularizer. [14] proposed a nonparametric estimation procedure for marked Hawkes processes and compared the results with other nonparametric methods. The study also establishes the relationship between the jump correlation matrix and the Hawkes kernel matrix using Wiener-Hopf systems of equations. [15] proposed a nonparametric approach where the research combines a parametric triggering effect and a nonparametric background intensity process, weighted kernel. By employing the “model-independent stochastic declustering algorithm (MISD)” to estimate both the background function and triggering effect [16]. A recent research by [17] expanded this approach to improve the estimation of the background intensity and enhance the capability of the trigger effect. In addition, [18] proposed a nonparametric approach for learning the triggering effect of the intensity function using online learning algorithm. This method makes use of the reproducing kernel Hilbert space for the triggering functions, and with proper regularization of the objective function, it becomes possible to reduce the triggering function estimation to an estimation of increasing sets of coefficients using the representer theorem. However, space-time self-exciting point process models are associated with high computational costs of estimation; therefore, fast algorithms are needed to enhance efficiency.

Criminology datasets provide rich insights into the timing and location of crime events, revealing complex dependencies between incidents. These dependencies often stem from individuals who report crimes, including direct participants, witnesses, or those indirectly informed about the events. Notably, a single criminal act can trigger a cascade of reports to law enforcement, creating a pronounced clustering of reports within a narrow time frame. By studying these datasets, researchers can uncover patterns in the dissemination of information related to criminal activities and identify hazard-prone areas. [19] introduced an Expectation-Maximization (EM)-type algorithm for maximizing the log-likelihood of a nonparametric model; however, it required substantial computation time to fit large datasets. Alternatively, a parametric model with exponential form of the triggering function spent less time than others when fitted to a large data set. Existing works often restrict parametric forms of the triggering functions to the exponential functions [20–22]. The intensity function can be assessed by considering only ‘recent’ occurrences, and the Markov property holds under exponential forms of the triggering functions. Although it is far less expensive computationally than using the nonparametric estimation, its applicability is limited.

In this paper, we focus on designing fast algorithm for estimating the intensity of self-exciting point process models specifically for crime events using data set from the city of Chicago, Illinois, in the United States, with an emphasis on a specific type of crime. For exploring the use of space-time self-exciting models for modeling crime datasets, a multi-dimensional Gaussian-type exponent approximation method based on the maximum likelihood (ML) is considered. The proposed nonparametric form allows for flexible estimation of the conditional intensity process in space-time self-exciting models and delves into space-time self-exciting point process models to better capture and characterize the dynamics of criminal events. The main problem is that the general approaches can be computationally expensive [18]. To solve the computation problem, a truncated conditional intensity process is proposed, which eases the computational difficulty that might be caused by the data size. By implementing the proposed method, we aim to examine the following: Is the proposed nonparametric approximation method both flexible and effective in handling space-time self-exciting point process models? Does the space-time self-exciting point process models based on the proposed method account significantly for the clustering characteristics observed in the crime datasets?

To address the primary concern, we consider simulation datasets generated from the crime model proposed by [3] to examine the efficiency of the developed nonparametric approach. The simulation-based algorithm introduced by [23] is used to generate the simulation datasets. For the second concern, we use residual analysis, a straightforward and effective method for model diagnostics [24], to evaluate the fitting performance. The contribution of this paper is to illustrate how point process analysis delivers valuable insight into the spread patterns of information during crime events and the distinctive factors of areas prone to hazards. And the proposed multi-dimensional Gaussian-type exponent approximation method can also be applied to other large datasets due to its flexibility in estimating the conditional intensity function and addressing computational challenges associated with large datasets.

In Section 2, we introduce the fundamental ideas of the space-time self-exciting point process models. Section 3 presents the multi-dimensional Gaussian-type exponent approximation method, the ML estimation, model diagnostics for the simulation algorithm and goodness-of-fit evaluation. In Section 4, we describe the effectiveness of the suggested method for fitting the simulated burglary datasets. The crime data set is analyzed in Section 5. In Section 6, a summary of the findings and potential avenues for further research would be provided.

## 2 Space-time self-exciting point process models

The space-time Hawkes point process is an extension of the univariate Hawkes process, generalizing it from the one-dimensional line X⊆ℝ to a higher-dimensional space where $X\subseteq {\mathbb{R}}^{2}\times \mathbb{R}$. Space-time Hawkes point process models incorporate the spatial component **s** into the univariate Hawkes process. These models were developed to simultaneously account for both spatial and temporal components when the scientific question of interest involves studying their joint behavior, something that cannot be addressed by separately analyzing spatial and temporal components of space-time data [25]. For instance, analyzing a series of burglary-related calls from residents over a period of time requires space-time data analysis, enabling the timely detection of localized spatial and temporal peaks in criminal activity. This approach helps identify burglary hotspots aiding in monitoring and crime prevention. The space-time point process is characterized via its conditional intensity λ(𝐬,t). By extending the conditional intensity function, the space-time point process models forecast event occurrences at spatial locations $𝐬\in X\subseteq {\mathbb{R}}^{2}$ and occurrence times t∈T⊆ℝ. In general, if the conditional intensity exists, it is not conditioned solely on the counting process *N*(**s**,*t*) but also the filtration $H_{t}$ which contains the history up to time *t*, given by $H_{t}=\{t_{i}:t_{i}<t\}$, but may include other additional information [6,26].

We denote *N* as a simple space-time point process in the interval *T* and within a two-dimensional spatial region $X\subseteq {\mathbb{R}}^{2}$. Let the filtration $H_{t}$ consist of all the historical events up to time *t*. The intensity process λ(𝐬,t) is interpreted as the average occurrence rate at which points cluster around a certain space-time location **s**, conditioned on the history $H_{t}$ of all points observed up to time *t*, where t∈T. This history includes all spatial locations and occurrence times of all events that happened before time *t*. Provided the conditional intensity function exists, we can defined it as in (1)


$$\lim_{△𝐬,△t\to 0}\frac{1}{△𝐬△t}P\{N([𝐬,𝐬+△𝐬)\times [t,t+△t)=1|H_{t}\}=\lambda (𝐬,t),$$ (1)

where the spatial coordinate is denoted as 𝐬:=(x,y)∈S.

Determining the influence of past variables on the conditional intensity is a major problem that needs to be addressed [27]. Modeling such point processes requires specifying unique mathematical structures for defining the conditional intensity function. Self-exciting point process models are a specific class of conditional intensity process models. For a space-time data set consisting of spatial locations $\left(x_{i},y_{i}\right)$ and occurrence times *t*_*i*_ up to time *t* for some events, the conditional intensity process of a space-time self-exciting point process is defined as follows.

For a simple space-time point process *N*, the conditional intensity function can be expressed as follows:


$$\begin{array}{cc} \lambda (𝐬,t|H_{t})= & \;\;\mu (𝐬,t)+\int_{S\times (-\infty ,t)}g(𝐬-\varepsilon ,t-\vartheta )\;N(d\varepsilon ,d\vartheta )\\ := & \;\;\mu (𝐬,t)+\sum_{i:t_{i}<t}g(𝐬-{𝐬}_{i},t-t_{i}), \end{array}$$ (2)

where $\left\{s_{1},s_{2},\ldots ,s_{n}\right\}$ represents the spatial locations of events, and $\left\{t_{1},t_{2},\ldots ,t_{n}\right\}$ denotes the corresponding occurrence times. For (𝐬,t)∈S×T, the function N(dε,dϑ) takes the value 1 if an infinitesimal element (dε,dϑ) contains an event $\left({𝐬}_{i},t_{i}\right)$ for some index *i*; otherwise, N(dε,dϑ) is equal to 0.

Equation (2) is partitioned into two categories: either a background intensity process (immigrant) or triggering effect (offspring or descendant). This is described by [26] as a subcritical (stationary) branching process with immigration. The background intensity process, μ(𝐬,t), defines the rate of incoming immigrants, independent of past events. New arrivals from background process are immigrants, while new arrivals triggered by past events are descendants. Whenever a new event occurs the total intensity λ(𝐬,t) increases due to self-excitation; however, the background intensity μ(𝐬,t) remains unchanged. For detailed introduction to self-exciting point processes and conditional intensity processes, see [28,29]. The log-likelihood of the conditional intensity process for the space-time point process model in (2), defined over the time frame $D:=[t_{*},t^{*})$, is given as follows. Following [29], let *N* be a space-time point process and assume that *n* points have been observed in the time interval $D:=[t_{*},t^{*})\subset T$. Define the spatial coordinates as ${𝐬}_{i}=(x_{i},y_{i})\in S\subseteq {\mathbb{R}}^{2}$. The log-likelihood function is given by:


$$\begin{array}{cc} \log L= & \;\;\int \int \int_{S\times D}\log\lambda (𝐬,t)\;N(d𝐬,dt)-\int \int \int_{S\times D}\lambda (𝐬,t)\;dxdydt\\ := & \;\;\sum_{i=1}^{n}\log\lambda ({𝐬}_{i},t_{i})-\int \int \int_{S\times D}\lambda (𝐬,t)\;dxdydt. \end{array}$$ (3)

It should be noted that the log-likelihood is contingent upon the selection of the observation period *D*. Additionally, the history $H_{t}$ described in (2) is modified to represent the sum of all events recorded within the time span $[t_{*},t),t\leq t^{*}$. It is very difficult to obtain the explicit form of the integral component of the log-likelihood function with the given model.

## 3 Methodology

In this section, we introduce a multi-dimensional Gaussian-type exponent approximation method for modeling the background and triggering components of a self-exciting point process. The estimation procedure and diagnostic methods are described in detail.

### Multi-dimensional Gaussian-type exponent approximation

The triggering function *g* and the background function μ are essential for characterizing a conditional intensity process. They shed light on how the trigger pattern in the observed data behaves in terms of space-time-dependent reliability. The space-time shape of the triggering function *g* can exhibit various forms, such as decreasing, increasin*g*, U-shaped, or inverted U-shaped patterns. When working with space-time point processes, it is often necessary to obtain accurate estimates of the background and triggering functions. Depending on the application, various parametric models, such as the ETAS model, can be employed to estimate these functions using the maximum likelihood approach. However, parametric models may be too restrictive, lacking the flexibility to capture the full range of triggering patterns. By contrast, nonparametric methods are more flexible and with adequate data and appropriate regularization, can yield consistent estimate of the triggering function. We propose a nonparametric method for estimating the conditional intensity function of a self-exciting point process.

### Triggering function

To model the self-exciting point process, we first introduce the self-excitation (triggering) function. For one dimensional self-exciting point process models, a generalized parametrization triggering function


$$g(t)=\sum_{j=0}^{q}\alpha_{j}e^{-\beta t}$$ (4)

was proposed by [30]. In this section we extend the one dimensional generalized parametrization triggering function to model space-time self-exciting models, explicitly accounting for spatial effects and broadening practical applicability. Considering that the spatial coordinates are unordered, we use quadratic term for space coordinates. A multi-dimensional Gaussian-type exponent approximation of the triggering function *g* is defined as follow:


$$\tilde{g}(x,y,t)=\sum_{j=0}^{q}b_{j}e^{-\alpha_{g,j}x^{2}-\beta_{g,j}y^{2}-\gamma_{g,j}t},$$ (5)

where $\int \int \int_{{\mathbb{R}}^{2}\times \mathbb{R}}\tilde{g}(x,y,t)\;dxdydt<1$, $b_{j}>0,\alpha_{g,j}>0,\beta_{g,j}>0,\gamma_{g,j}>0,j=0,\ldots ,q$. $\alpha_{g,j}$ and $\beta_{g,j}$ are the triggering spatial decay rates along the *x* and *y* axes, and $\gamma_{g,j}$ is the temporal decay rate.

Extensive research has been conducted on the application of a single isotropic “Gaussian kernel” for locations and an exponential kernel for the temporal coordinate in seismicity, crime studies, security, and social network datasets [3,5,22]. We propose the multi-dimensional Gaussian-type exponent approximation to expand the “Gaussian kernel” for spatial coordinates from single component to multiple components, and use the combine multi-component “Gaussian kernel” for spatial coordinates with exponential kernel for time coordinate. Then the proposed multi-dimensional Gaussian-type exponent approximation can be seen as a mixed model.

### Stationary background rate

The application of self-exciting point processes mostly assumes that the background intensity μ is considered to be time-invariant, meaning it remains constant over time [2,6,15,16,18]. In our approach, we adopt a similar estimation method while considering a stationary background intensity process for the space-time point process models, where the baseline intensity depends on the spatial component (*x*,*y*) but not on temporal component *t*, this implies that the background intensity exhibits spatial variation but is temporally invariant:


$$\tilde{\mu }(x,y)=\sum_{j=0}^{p}a_{j}e^{-\alpha_{\mu ,j}(x-c_{j}{)}^{2}-\beta_{\mu ,j}(y-d_{j}{)}^{2}},$$ (6)

where $a_{j}>0,c_{j}>0,d_{j}>0,\alpha_{\mu ,j}>0,\beta_{\mu ,j}>0,\;j=0,\ldots ,p$.

Note that $\tilde{\mu }$ has mean parameters, but $\tilde{g}$ does not. The *c*_*j*_ and *d*_*j*_ represents the mean parameters in the background intensity. This is because, for the background function μ, it may be centered at any location, and we need to estimate the center; for the triggering function *g*, the center is the location of the parent event, we do not need to estimate the center. $\alpha_{\mu ,j}$ and $\beta_{\mu ,j}$ are the background spatial decay rates along the *x* and *y* axes, respectively.

**Remark 1** *The current model can be extended to cases where covariance differentials (space correlation) are incorporated into both the background and the triggering function. That is,*


μ(x,y)=αu(x,y)

*and*


$$u(x,y)\sim \sum_{k=1}^{k_{1}}p_{u,k}𝒩(x_{i},y_{i}|\mu_{u,k},\Sigma_{u,k}).$$

*Triggering function expressed as*


g(x,y,t)=βh(x,y,t)

*and*


$$h(x,y,t)\sim \sum_{j=1}^{k_{2}}p_{h,j}𝒩(x_{i},y_{i},t_{i}|\mu_{h,j},\Sigma_{h,j}),$$

*where* $\Sigma_{u,k}$ *and* $\Sigma_{h,j}$ *are the covariance matrices, and* $\mu_{u,k}$ *and* $\mu_{h,j}$ *are the means of the background and triggering functions, respectively. However, the above extension makes the design of fast estimation algorithms challenging. We will investigate this extended model in subsequent studies. It is worth noting that although the method proposed in this paper does not account for spatial correlations, the simulation results demonstrate that the proposed model and estimation method still exhibit robust performance for datasets involving spatial correlation.*

### Estimation methods

With the background and triggering functions defined above, we proceed to estimate the model parameters using MLE. In most cases, explicit solutions for MLE are not available and iterative numerical optimization methods are used instead. Alternatively, [26] showed that the EM algorithm can efficiently optimize the log-likelihood function in parametric models. The EM-type methods were first introduced in [15] for a semi-parametric model and have since been frequently applied to both parametric and nonparametric models in estimating the background intensity function and the triggering effects [3,16,17,22,23,31,32]. In this section, we address the computational difficulties associated with existing research and the challenges encountered when dealing with large datasets. We introduce a fast estimation algorithm for the space-time self-exciting point process model in two dimensions. First, we derive the explicit form of the integral component of the log-likelihood function; second, we introduce a truncated intensity to accelerate the computation.

The proposed multi-dimensional Gaussian-type exponent approximation method, described in the previous section, enhances the flexibility of intensity function estimation and alleviates the computational barriers that make space-time point process models challenging and computationally expensive to handle, especially when dealing with large datasets. Based on (3), for a defined spatial region $S:=[x_{*},x^{*}]\times [y_{*},y^{*}]$ and a defined time window $D:=[t_{*},t^{*})$, where $S\subseteq {\mathbb{R}}^{2}$ and D⊆ℝ. The log-likelihood is given as follows:


$$\log L=\sum_{i=1}^{n}\log\lambda (x_{i},y_{i},t_{i})-\int \int \int_{S\times D}\lambda (x,y,t)\;dxdydt.$$ (7)

We obtain the MLE of the conditional intensity function by maximizing the log-likelihood (7).

The direct computation using (7) is computationally expensive; therefore, we segment and compute each part of the equation to reduce it computational burden. The first part of the log-likelihood in (7), which represents the log of the intensity function, is segmented into the background and the triggering effect. The computation is performed separately to mitigate computational challenges. The integral part of (7) is also divided into two segments, both represented via a Gaussian distribution. This segmentation accelerates the computation process and reduce the computational burden.

Dealing with the integral (compensator) term of the log-likelihood in (7), we first evaluate the background intensity in (6). Let Φ(x,μ,σ) represent a Gaussian distribution value at *x* where the Gaussian distribution has mean μ and standard deviation σ. Since


$$\begin{array}{c} \int_{x_{*}}^{x^{*}}e^{-\alpha_{\mu ,j}(x-c_{j}{)}^{2}}dx=\frac{\sqrt{\pi }}{\sqrt{\alpha_{\mu ,j}}}[\Phi (x^{*},c_{j},\sqrt{\frac{1}{2\alpha_{\mu ,j}}})-\Phi (x_{*},c_{j},\sqrt{\frac{1}{2\alpha_{\mu ,j}}})],\\ \int_{y_{*}}^{y^{*}}e^{-\beta_{\mu ,j}(y-d_{j}{)}^{2}}dy=\frac{\sqrt{\pi }}{\sqrt{\beta_{\mu ,j}}}[\Phi (y^{*},d_{j},\sqrt{\frac{1}{2\beta_{\mu ,j}}})-\Phi (y_{*},d_{j},\sqrt{\frac{1}{2\beta_{\mu ,j}}})], \end{array}$$

we obtain


$$\begin{array}{cc} \int_{t_{*}}^{t^{*}}\int_{y_{*}}^{y^{*}}\int_{x_{*}}^{x^{*}}e^{-\alpha_{\mu ,j}(x-c_{j}{)}^{2}-\beta_{\mu ,j}(y-d_{j}{)}^{2}}\;dxdydt= & \;\frac{\pi }{\sqrt{\alpha_{\mu ,j}\beta_{\mu ,j}}}[\Phi (x^{*},c_{j},\sqrt{\frac{1}{2\alpha_{\mu ,j}}})\\ - & \;\Phi (x_{*},c_{j},\sqrt{\frac{1}{2\alpha_{\mu ,j}}})]\\ & \;\times [\Phi (y^{*},d_{j},\sqrt{\frac{1}{2\beta_{\mu ,j}}})\\ & \;-\Phi (y_{*},d_{j},\sqrt{\frac{1}{2\beta_{\mu ,j}}})](t^{*}-t_{*}). \end{array}$$

Next, we evaluate the triggering effect (5) of the intensity process. Notice that


$$\begin{array}{cc} \int_{x_{*}}^{x^{*}}e^{-\alpha_{g,j}x^{2}}dx & \;=\frac{\sqrt{\pi }}{\sqrt{\alpha_{g,j}}}[\Phi (x^{*},x_{i},\sqrt{\frac{1}{2\alpha_{g,j}}})-\Phi (x_{*},x_{i},\sqrt{\frac{1}{2\alpha_{g,j}}})],\\ \int_{y_{*}}^{y^{*}}e^{-\beta_{g,j}y^{2}}dy & \;=\frac{\sqrt{\pi }}{\sqrt{\beta_{g,j}}}[\Phi (y^{*},y_{i},\sqrt{\frac{1}{2\beta_{g,j}}})-\Phi (y_{*},y_{i},\sqrt{\frac{1}{2\beta_{g,j}}})],\\ \int_{t_{*}}^{t^{*}}e^{-\gamma_{g,j}t}dt & \;=\frac{1}{\gamma_{g,j}}e^{-\gamma_{g,j}(t_{i}-t^{*})} \end{array}$$

and


$$\begin{array}{cc} \int_{t_{*}}^{t^{*}}\int_{y_{*}}^{y^{*}}\int_{x_{*}}^{x^{*}}e^{-\alpha_{g,j}x^{2}-\beta_{g,j}y^{2}-\gamma_{g,j}t}\;dx\;dy\;dt= & \;\frac{\pi }{\sqrt{\alpha_{g,j}\beta_{g,j}}}[\Phi (x^{*},x_{i},\sqrt{\frac{1}{2\alpha_{g,j}}})\\ & \;-\Phi (x_{*},x_{i},\sqrt{\frac{1}{2\alpha_{g,j}}})]\\ & \;\;\;\times [\Phi (y^{*},y_{i},\sqrt{\frac{1}{2\beta_{g,j}}})\\ & \;-\Phi (y_{*},y_{i},\sqrt{\frac{1}{2\beta_{g,j}}})][\frac{1}{\gamma_{g,j}}e^{-\gamma_{g,j}(t_{i}-t^{*})}]. \end{array}$$

Then we have


$$\begin{array}{cc} L & \;=\sum_{i=1}^{n}\log[\mu (x_{i},y_{i})+\sum_{j=0}^{q}b_{j}G_{j}(i)]\\ & \;\;\;-\sum_{j=0}^{p}a_{j}A_{j}-\sum_{i=1}^{n}\sum_{j=0}^{q}b_{j}B_{j}(i)H_{j}(t^{*}-t_{i}), \end{array}$$

where $H_{j}(t)=(1-e^{-\gamma_{g,j}t})\gamma_{g,j}^{-1}$, *G*_0_(1)=0,


$$G_{j}(i)=\int_{S\times [t_{*},t_{i})}e^{-\alpha_{g,j}(x_{i}-u{)}^{2}-\beta_{g,j}(y_{i}-v{)}^{2}-\gamma_{g,j}(t_{i}-s)}\;N(du,dv,ds),\;i>1,$$

$$\begin{array}{cc} A_{j} & \;=\frac{\pi }{\sqrt{\alpha_{\mu ,j}\beta_{\mu ,j}}}[\Phi (x^{*},c_{j},\sqrt{\frac{1}{2\alpha_{\mu ,j}}})-\Phi (x_{*},c_{j},\sqrt{\frac{1}{2\alpha_{\mu ,j}}})]\\ & \;\;\;\times [\Phi (y^{*},d_{j},\sqrt{\frac{1}{2\beta_{\mu ,j}}})-\Phi (y_{*},d_{j},\sqrt{\frac{1}{2\beta_{\mu ,j}}})](t_{n}-t) \end{array}$$

and


$$\begin{array}{cc} B_{j}(i) & \;=\frac{\pi }{\sqrt{\alpha_{g,j}\beta_{g,j}}}[\Phi (x^{*},x_{i},\sqrt{\frac{1}{2\alpha_{g,j}}})-\Phi (x_{*},x_{i},\sqrt{\frac{1}{2\alpha_{g,j}}})]\\ & \;\;\;\times [\Phi (y^{*},y_{i},\sqrt{\frac{1}{2\beta_{g,j}}})-\Phi (y_{*},y_{i},\sqrt{\frac{1}{2\beta_{g,j}}})]. \end{array}$$

To reduce the computational burden of *G*_*j*_(*i*), we make the model more tractable by assuming that an event occurring at time *t*_*i*_ and spatial location **s**_*i*_ influences the intensity process λ(x,y,t) by an amount $g(x-x_{i},y-y_{i},t-t_{i})$ within a window of $i\in \{i:t_{i}\in [t-z,t)\}$, where *z* is the truncated time bound. Under certain assumptions, such as *g*(*x*,*y*,*t*) tends to 0 fast, which is a weak restriction and satisfies most application situations, the approximation error due to truncation can be effectively controlled [18]. We have the following concept of “truncated intensity”, which belongs to [18]:


$$\begin{array}{cc} \lambda^{\left(z\right)}(x,y,t) & \;:=\mu (x,y)+\int_{S\times [0,t)}1_{\left\{t-u\leq z\right\}}g(x-u,y-v,t-s)\;N(du,dv,ds)\\ & \;\;=\mu (x,y)+\sum_{i:t-z\leq t_{i}<t}g(x-x_{i},y-y_{i},t-t_{i}). \end{array}$$ (8)

However, the summation part of (8) for computing $\lambda^{\left(z\right)}(x_{k},y_{k},t_{k})$ is irregular, so we replace $\left\{i:t-z\leq t_{i}<t\right\}$ with fixed number of summation for each *i* by $n^{*}:=\max\{\#\{i:t-z\leq t_{i}<t\},i=1,\ldots ,n\}$. Then we have the modified truncated intensity function:


$$\lambda^{\left(z\right)}(x_{k},y_{k},t_{k})=\mu (x_{k},y_{k})+\sum_{j=k-n^{*}}^{k-1}g(x_{k}-x_{j},y_{k}-y_{j},t_{k}-t_{j}).$$ (9)

Then the computational complexity of summation part in (9) at *n* points reduces from *O*(*n*^2^) to $O(n^{*}n)$. Now we can approximate *G*_*j*_(*i*), i=2,3,…,n as follows:


$${\tilde{G}}_{j}(i)=\int_{S\times [\max\{t_{*},t_{i-n^{*}}\},t_{i})}e^{-\alpha_{g,j}(x_{i}-u{)}^{2}-\beta_{g,j}(y_{i}-v{)}^{2}-\gamma_{g,j}(t_{i}-s)}\;N(du,dv,ds).$$

Obtaining MLE in space-time self-exciting point process models is particularly challenging to accomplish analytically, as the structure of the log-likelihood in (7) consists of a sum of logarithms of background and triggering functions, which involves summing over previous points, resulting in intractable analytical maximization [33]. Hence we use the nonlinear optimization procedure described in [5] to obtain the MLE.

### Selection *p* and *q*

To compare the adequacy of the proposed models using goodness-of-fit for different *p* and *q*, we use the Bayes Information Criterion (BIC):


$$\begin{array}{cc} \text{BIC} & \;=-2\max(\ln(L))+(\text{number of adjusted parameters})\ln(n)\\ & \;=-2\max(\ln(L))+(5(p+1)+4(q+1))\ln(n). \end{array}$$ (10)

A self-exciting model with the smallest BIC value is considered the best fit.

### Diagnostics

To evaluate the performance of point process models, residual analysis is commonly employed. Various diagnostic techniques have been proposed for self-exciting point process models [24,34–36]. In this study, we employ residual analysis using super-thinning introduced by [37] for both simulations and the Chicago crime data analysis. The thinning method introduced by [24] is simple, efficient, and possesses the characteristics that any process governed by its conditional intensity process can be reduced by thinning to yield a homogeneous Poisson process [30]. However, residuals obtained through thinning can face challenges such as variability and the lack of independence among the residual points after thinning, primarily as a result of losing information caused by the elimination of observed points. To address these challenges, [37] proposed the super-thinning which merges the observed points obtained through thinning with simulated points generated by superposition. This approach produces a homogeneous residual point process, provided that the conditional intensity is correctly estimated. The procedure outlined below follows a thinning algorithm introduced by [24]:

**Algorithm 1 Thinning algorithm**

(1) Start by defining $b=\inf_{x,y,t}\hat{\lambda }(x,y,t)$;

(2) For every observed event *i*, compute the value $p_{i}=\frac{b}{\hat{\lambda }(x_{i},y_{i},t_{i})}$;

(3) Event *i* is retained with probability *p*_*i*_.

For the application of super-thinning, since *b* is small, selecting the rate *k* as $b=\inf_{x,y,t}\hat{\lambda }(x,y,t)$ will result in few points after thinning, rendering the test uninformative. Therefore, we select the rate *k* such that $b\leq k\leq \sup_{x,y,t}\hat{\lambda }(x,y,t)$. Using the alternative method suggested by [37], we choose the value of *k* to optimize the effectiveness of formal tests in assessing the homogeneity of the residuals,


$$k=\frac{1}{\left|S||D\right|}\int \int \int_{S\times D}\hat{\lambda }(x,y,t)\;dtdxdy,$$

where |*S*| denote the area of the spatial domain S and |*D*| is the length of time within the interval *D*. We first thin the process with probabilities $p_{i}=\min\{k/\hat{\lambda }(x_{i},y_{i},t_{i}),1\}$, then we add to the thinned process inhomogeneous Poisson process with intensity $\max\left\{k-\hat{\lambda }(x,y,t),0\right\}$. This will result in a homogenous point process with rate *k* if the conditional intensity $\hat{\lambda }(x,y,t)$ is correctly estimated.

Ripley’s *K*-function [38] determines the proportion of occurrences per unit area within a specific distance and can be used for model diagnostics in super-thinning. This helps assess whether the model still fails to account for clustering in the thinning process [24]. Below is the most frequently applied *K*-function incorporating edge-corrected estimators [38]:


$$\hat{K}(d)=|S|n^{-2}\sum_{i}\sum_{j\neq i}w({𝐬}_{i},{𝐬}_{j}{)}^{-1}1_{d_{ij}<d},$$ (11)

where the weight function $w({𝐬}_{i},{𝐬}_{j})$ corresponds to the proportion of the circle’s circumference that lies within the study area, *d*_*ij*_ denotes the distance between the *i*th and *j*th points, and |S| represents the volume of the observation region. In comparison to Ripley’s *K*-function, the *L*-function, computed as $L(d)=\sqrt{\hat{K}(d)/\pi }$, exhibits more consistent variance. Hence, we employ the *L*-function for model diagnostics. Based on Monte Carlo techniques and the *L*-function, the following algorithm provides the diagnostic procedure.

### Diagnostics algorithm

Start with a tuning parameter *k*, $\hat{\lambda }(x,y,t)$, which represents the estimated intensity, *M*, which denotes the number of simulations and a discrete value *d*. We follow these steps:

**Algorithm 2 Super-thinning diagnostic algorithm**

(1) Use super-thinning approach to derive the thinned process;

(2) Compute the *L*-functions;

(3) Perform Steps 1 and 2 iteratively for *M* repetitions;

(4) Compute the sample mean of *L*-functions;

(5) Generate a homogeneous Poisson process with a rate of *k* across the observed domain *S* and repeat Step 2 *M* times;

(6) Based on the *M* realizations of the homogeneous Poisson process, calculate the 95% confidence limits for the *L*-functions.

## 4 Numerical experiments

### Synthetic datasets

This section focuses on evaluating the efficiency of the proposed method by generating artificial burglary datasets from the conditional intensity process (12) with approximate sizes $5\times 10^{3}$ and $5\times 10^{4}$. [3] considered a conditional-intensity process (12) and reported results consistent with patterns observed in real burglary data. We incorporate a correlation coefficient, ρ, into our simulation model to capture dependence between the *x* and *y* coordinates. The background intensity is modeled as a correlated bivariate Gaussian density scaled by $\overset{―}{\mu }$, whereas the triggering intensity is modeled as the product of a correlated bivariate Gaussian spatial kernel and an exponential temporal kernel. By varying ρ, we evaluate the proposed model’s performance on datasets generated by this simulation framework.


$$\left\{ \begin{array}{c} \mu (x,y,t)=\frac{\overset{―}{\mu }}{(2\pi )(\sigma_{\mu }{)}^{2}}\exp\{\frac{1}{1-\rho^{2}}(-\frac{(x-c{)}^{2}}{2\sigma_{\mu }^{2}}-\frac{(y-d{)}^{2}}{2\sigma_{\mu }^{2}}+\frac{\rho (x-c)(y-d)}{(\sigma_{\mu }{)}^{2}})\},\\ g(x,y,t)=\frac{\theta \omega }{2\pi \sigma_{x}\sigma_{y}}\exp(-\omega t)\exp\{\frac{1}{1-\rho^{2}}(-\frac{x^{2}}{2\sigma_{x}^{2}}-\frac{y^{2}}{2\sigma_{y}^{2}}+\frac{\rho xy}{\sigma_{x}\sigma_{y}})\}, \end{array}\right.$$ (12)

where $\overset{―}{\mu }=5.71$, $\sigma_{\mu }=4.5$, *c* = 10, *d* = 10, θ=0.2, $\omega^{-1}=10$, $\sigma_{x}=0.01$ and $\sigma_{y}=0.1$. In a spatial domain of 20 × 20, the simulation is conducted utilizing the **Simulation Algorithm** introduced by [23]. To ensure that the point process reaches a steady state, discard the first and last 2000 points in each simulation [3]. The above simulation function only select single component. To illustrate the multi-component for the simulation, we use the following model where *p* = 0 and *q* = 1:


$$\begin{array}{c} \left\{ \begin{array}{cc} \mu (x,y,t)= & \frac{\overset{―}{\mu }}{(2\pi )(\sigma_{\mu }{)}^{2}}\exp\{\frac{1}{1-\rho^{2}}(-\frac{(x-c{)}^{2}}{2\sigma_{\mu }^{2}}-\frac{(y-d{)}^{2}}{2\sigma_{\mu }^{2}}+\frac{\rho (x-c)(y-d)}{(\sigma_{\mu }{)}^{2}})\},\\ g(x,y,t)= & \frac{\theta_{1}\omega_{1}}{2\pi \sigma_{x1}\sigma_{y1}}\exp(-\omega_{1}t)\exp\{\frac{1}{1-\rho^{2}}(-\frac{x^{2}}{2\sigma_{x1}^{2}}-\frac{y^{2}}{2\sigma_{y1}^{2}}+\frac{\rho xy}{\sigma_{x1}\sigma_{y1}})\}\\ & +\frac{\theta_{2}\omega_{2}}{2\pi \sigma_{x2}\sigma_{y2}}\exp(-\omega_{2}t)\exp\{\frac{1}{1-\rho^{2}}(-\frac{x^{2}}{2\sigma_{x2}^{2}}-\frac{y^{2}}{2\sigma_{y2}^{2}}+\frac{\rho xy}{\sigma_{x2}\sigma_{y2}})\}, \end{array}\right. \end{array}$$ (13)

where $\overset{―}{\mu }=5.71$, $\sigma_{\mu }=4.5$, *c* = 10, *d* = 10, $\theta_{1}=0.2$, $\omega_{1}^{-1}=10$, $\sigma_{x1}=0.01$, $\sigma_{y1}=0.1$, $\theta_{2}=0.3$, $\omega_{2}^{-1}=20$, $\sigma_{x2}=0.2$ and $\sigma_{y2}=0.05$.

### Fitting synthetic datasets

Moving forward, after generating the artificial burglary datasets using the simulation algorithm, we fit them to the model in (9). Considering the tuning parameter, which is the length of the truncated intensity function *z* in (9), we select *z* from the range 100–1000. This choice is based on the observation that a small value of *z* causes the background function to be overestimated, whereas a large value of *z* makes the estimation computationally expensive. Therefore, we found 200 to be the appropriate truncation length.

**Fig 1:**
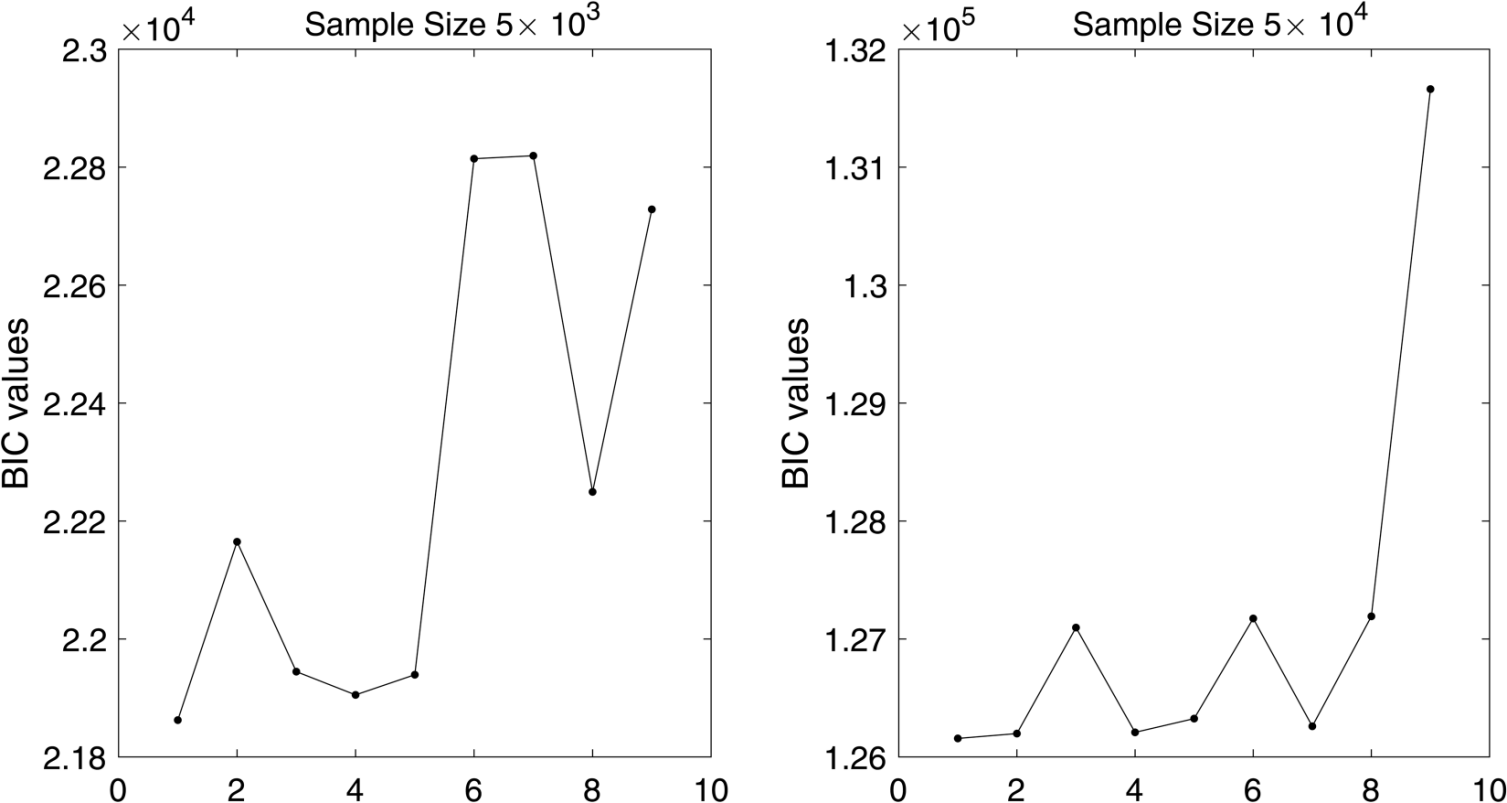
BIC values with different data sizes when ρ=0 for single component case.

**Fig 2:**
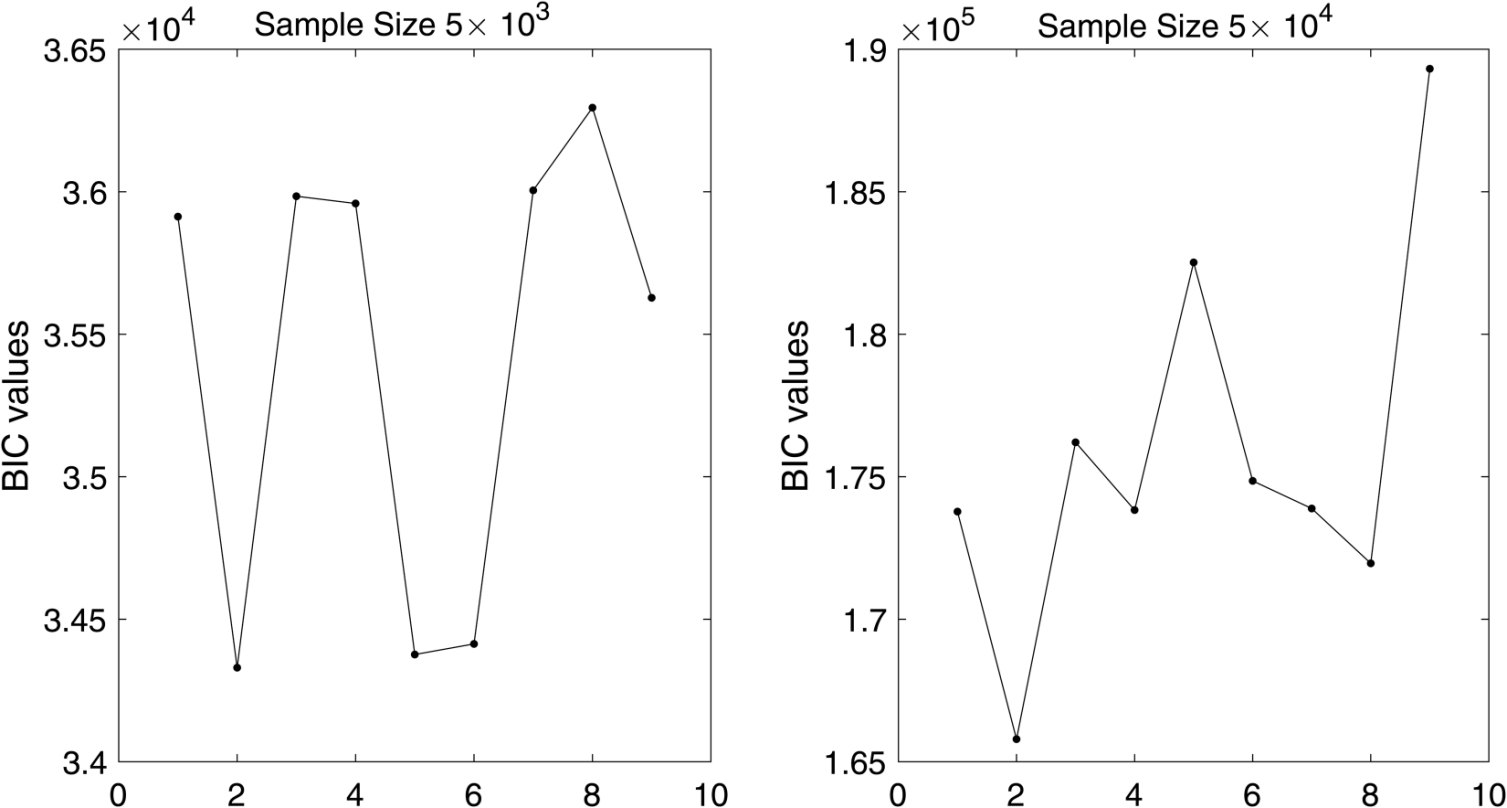
BIC values with different data sizes when ρ=0 for multi-component case.

In Figs 1 and 2, we plot the values of BIC(*i*) for i=1,2,…,9, corresponding to


(p,q)∈{(0,0),(0,1),(0,2),(1,0),(1,1),(1,2),(2,0),(2,1),(2,2)},

for the single and the multi-component cases, respectively. We observe that (*p*,*q*) = (0,0) yields the smallest BIC value for both datasets across all ρ values under the single component specification as shown in Fig 1, whereas in the multi-component setting the selected order is (*p*,*q*)=(0,1) for both datasets as illustrated in Fig 2.

**Fig 3:**
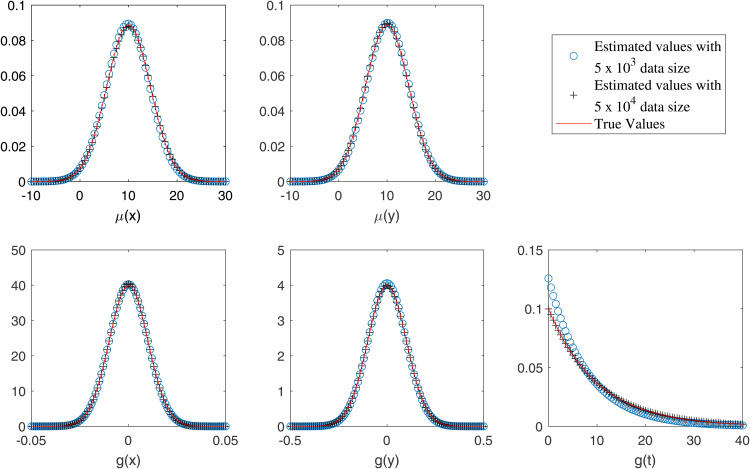
Estimated (circles and pluses) and actual (solid line) marginals when ρ=0 for single component case.

**Fig 4:**
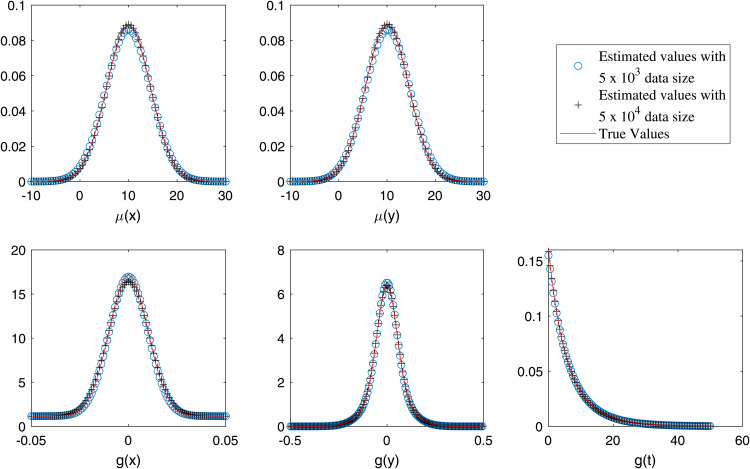
Estimated (circles and pluses) and actual (solid line) marginals when ρ=0 for multi-component case.

In Tables 1 and 2, we present the estimated and exact parameter values for datasets of approximately $5\times 10^{3}$ and $5\times 10^{4}$ events, respectively, under varying values of ρ. The results suggest that the estimated parameter values are closely aligned with the corresponding exact values, although minor discrepancies exist. [39] demonstrated that such estimations are prone to bias and proposed the use of Laplace approximation to mitigate it, although some bias still remains. Similarly, we report parameter estimates for the multi-component case using (13). The bias becomes more pronounced when ρ>0, as illustrated for ρ=0.05and0.1 in Tables 3 and 4. In Fig 3, we plot the marginal estimates of μ(x,y,t) and *g*(*x*,*y*,*t*) against their corresponding true distributions. The estimated marginals are shown to closely follow the true marginals. Additionally, we present a comparison of the true and estimated marginals of μ(x,y,t) and *g*(*x*,*y*,*t*) for the multi-component scenario in Fig 4. Although the proposed model excludes the spatial correlation parameter ρ, it still yields parameter estimates close to the true values on datasets generated with spatial correlation. In Tables 5 and 6, we assess the robustness of our proposed method in the single component (single) and multi-component (multi) settings using 50 simulated datasets per setting.

**Table 1:** Parameter value estimates for $5\times 10^{3}$data size.

ρ	Parameters	$\overset{―}{\mu }$	$\sigma_{\mu }$	*c*	*d*	ω	θ	$\sigma_{x}$	$\sigma_{y}$
0.00	True values	5.71	4.5	10	10	0.1	0.2	0.01	0.1
	Estimated values	5.7927/5.7277	4.4582/4.4332	9.8957	10.1285	0.1258	0.1910	0.0099	0.0983
0.10	Estimated values	5.7807/5.7296	4.5018/4.4818	9.9126	10.0823	0.1224	0.1884	0.0102	0.0971
0.20	Estimated values	5.6926/5.5451	4.4860/4.4274	9.9320	10.0676	0.1214	0.1847	0.0099	0.1022
0.30	Estimated values	5.6043/5.4543	4.4994/4.4388	9.9178	10.0831	0.1196	0.1960	0.0098	0.1013
0.40	Estimated values	5.3543/5.3075	4.5020/4.4823	9.9629	10.1449	0.1234	0.1960	0.0101	0.0966

**Table 2:** Parameter value estimates for $5\times 10^{4}$ data size.

ρ	Parameters	$\overset{―}{\mu }$	$\sigma_{\mu }$	*c*	*d*	ω	θ	$\sigma_{x}$	$\sigma_{y}$
0.00	True values	5.71	4.5	10	10	0.1	0.2	0.01	0.1
	Estimated values	5.7629/5.6384	4.5308/4.4816	9.9804	10.0125	0.1007	0.1987	0.0100	0.0984
0.10	Estimated values	5.6928/5.6126	4.5097/4.4779	9.9843	9.9972	0.1004	0.1978	0.0101	0.0992
0.20	Estimated values	5.5909/5.5629	4.4990/4.4877	9.9896	9.9865	0.1026	0.1963	0.0100	0.0991
0.30	Estimated values	5.4459/5.3920	4.4858/4.4636	9.9716	10.0115	0.1005	0.1948	0.0100	0.0978
0.40	Estimated values	5.2039/5.1762	4.4515/4.4396	9.9918	10.0059	0.0991	0.1970	0.0099	0.0990

**Table 3:** Parameter value estimates for $5\times 10^{3}$ data size.

ρ	Parameters	$\overset{―}{\mu }$	$\sigma_{\mu }$	*c*	*d*	$\omega_{1}$	$\theta_{1}$	$\sigma_{x1}$	$\sigma_{y1}$	$\omega_{2}$	$\theta_{2}$	$\sigma_{x2}$	$\sigma_{y2}$
0.0	True values	5.71	4.5	10	10	0.1	0.2	0.01	0.1	0.2	0.3	0.2	0.05
	Estimated values	5.8364/5.8412	4.6343/4.6362	9.9394	10.1362	0.1027	0.1929	0.0100	0.0985	0.1890	0.2963	0.2046	0.0491
0.05	Estimated values	5.9464/6.1028	4.5850/4.6449	9.9094	10.1367	0.0004	0.0663	20435.0957	33.3791	0.1464	0.4709	0.1478	0.0669
0.1	Estimated values	5.9531/5.8954	4.6113/4.5889	9.9120	10.0985	0.1476	0.1574	0.1420	280.8152	1.7194	0.4720	17.0684	0.0769

**Table 4:** Parameter value estimates for $5\times 10^{4}$ data size.

ρ	Parameters	$\overset{―}{\mu }$	$\sigma_{\mu }$	*c*	*d*	$\omega_{1}$	$\theta_{1}$	$\sigma_{x1}$	$\sigma_{y1}$	$\omega_{2}$	$\theta_{2}$	$\sigma_{x2}$	$\sigma_{y2}$
0.0	True values	5.71	4.5	10	10	0.1	0.2	0.01	0.1	0.2	0.3	0.2	0.05
	Estimated values	5.7471/5.7288	4.4776/4.4705	9.9985	10.0575	0.1052	0.2009	0.0105	0.0968	0.1945	0.2992	0.2008	0.0503
0.05	Estimated values	5.8268/5.7524	4.4861/4.4574	10.0304	10.0389	0.1370	0.0033	6.7116	48671.6307	0.1463	0.4927	0.1343	0.0888
0.1	Estimated values	5.9755/5.8829	4.4884/4.4535	10.0100	10.0656	0.1520	0.0934	0.1421	183.2325	0.2533	0.4809	800.7645	0.0687

**Table 5:** Mean Parameter estimates and Variances for $5\times 10^{3}$ data size across 50 simulations when ρ=0.

	Parameters	$\overset{―}{\mu }$	*σ* *μ*	*c*	*d*	*ω*1	*θ*1	*σ*x1	*σ*y1	*ω*2	*θ*2	*σ*x2	*σ*y2
Single	True values	5.71	4.5	10	10	0.1	0.2	0.01	0.1				
	Mean	5.8152/5.8212	4.5041/4.5066	9.9914	10.0141	0.1225	0.1882	0.0100	0.0996				
	Variance	0.0404/0.0327	0.0056/0.0047	0.0061	0.0070	1.5×10−5	3.1×10−5	7.4×10−8	9.8×10−6				
Multi	True values	5.71	4.5	10	10	0.1	0.2	0.01	0.1	0.2	0.3	0.2	0.05
	Mean	5.7930/5.7857	4.5089/4.5062	9.9782	9.9944	0.1060	0.1943	0.0177	0.1017	0.1978	0.3029	0.1987	0.0500
	Variance	0.0278/0.0172	0.0050/0.0028	0.0051	0.0072	4.3×10−5	0.0008	0.0028	0.0002	9.3×10−5	0.0007	7.7×10−5	7.2×10−6

**Table 6:** Mean Parameter estimates and Variances for $5\times 10^{4}$ data size across 50 simulations with ρ=0.

	Parameters	$\overset{―}{\mu }$	*σ* *μ*	*c*	*d*	*ω*1	*θ*1	*σ*x1	*σ*y1	*ω*2	*θ*2	*σ*x2	*σ*y2
Single	True values	5.71	4.5	10	10	0.1	0.2	0.01	0.1				
	Mean	5.7996/5.8011	4.5037/4.5043	9.9980	10.0008	0.1220	0.1880	0.00998	0.0996				
	Variance	0.0041/0.0054	0.0007/0.0011	0.0013	0.0010	1.6×10−6	6.1×10−6	1.3×10−8	1.7×10−6				
Multi	True values	5.71	4.5	10	10	0.1	0.2	0.01	0.1	0.2	0.3	0.2	0.05
	Mean	5.7686/5.7583	4.5014/4.4974	9.9941	9.9995	0.1050	0.1874	0.0398	0.1150	0.1971	0.3087	0.2000	0.0583
	Variance	0.0053/0.0059	0.00084/0.00075	0.0013	0.0011	0.00021	0.0048	0.0094	0.0039	0.00037	0.0044	0.00088	0.0023

**Fig 5:**
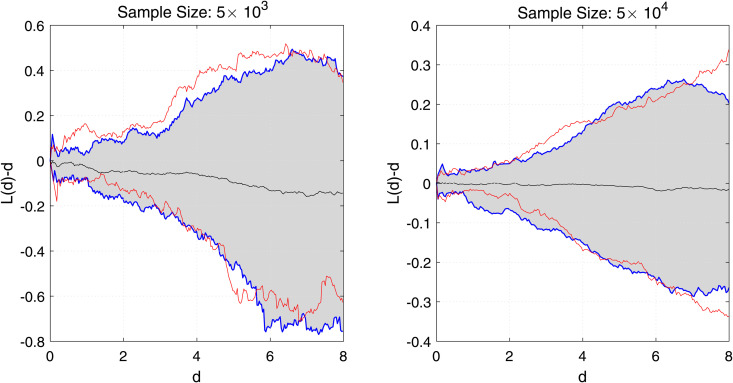
Super-thinning residuals across different data sizes for the single component scenario. The lower red curve represents the 5% bounds of the estimated centered *L*-function, L(d)−d, for a homogeneous Poisson process, while the upper red curve corresponds to its 95% bounds. Similarly, the lower blue curve denotes the 5% bounds of the estimated centered *L*-function for the thinned residuals, and the upper blue curve indicates its 95% bounds. The middle black line represents the empirical mean of the estimated centered *L*-function for the thinned residuals, while the gray-shaded region illustrates the confidence interval of the estimated centered *L*-function for the thinned residuals.

Fig 5 presents the estimated centered *L*-function, L(d)−d, along with the 95% confidence limits for homogeneous Poisson processes. When the difference between the *L*(*d*) and *d* is zero, it implies homogeneity, whereas any deviation from zero indicates heterogeneity. As shown in Fig 5, the thinned residuals appear to be homogeneous, as the estimated centered *L*-function remains within the 95% confidence bounds.

For Figs 1 and 2, the order of the proposed model is selected using the BIC criterion, which plays a role analogous to bandwidth selection in kernel density estimation (KDE)-based methods that rely on cross-validation [3,19]. The proposed model selection procedure is computationally efficient compared to KDE-based approaches, which are considerably more expensive as they require repeated cross-validation. From Fig 3, the results indicate that for a dataset of size $5\times 10^{3}$, the estimates are reasonable, while the variability decreases as the data size increases to $5\times 10^{4}$, with similar behavior observed in Fig 4. These observations are consistent with the results of [3,40]. This indicates that, with an appropriate choice of model order, the method avoids model misspecification, leading the proposed approach to closely approximate the true marginals when ρ=0. Furthermore, following the residual analysis approaches introduced in the literature [6,37,40,41], the results in Fig 5 indicate that the model passes the goodness-of-fit test for both datasets. This implies that the test effectively evaluates the proposed model.

In summary, Tables 1–6 show that the proposed estimation framework provides stable and reliable parameter recovery in both single and multi-component settings. The estimated parameters closely align with the true values across different sample sizes, with discrepancies decreasing as the number of events increases. While a slight bias is observed in the presence of spatial correlation, the method reliably captures the marginal structures of μ(x,y,t) and *g*(*x*,*y*,*t*). In the multi-component case, this bias becomes more evident when ρ>0. Overall, these results affirm the robustness of the proposed approach across a range of simulation scenarios.

## 5 Crime data set analysis

### Data

In this section of our study, we analyze burglary crime data set in Chicago, United States, during the year 2002. We use the proposed approach to fit the burglary data set compiled by Chicago police department. The city of Chicago is the largest city in Illinois and the Midwest, with a population of 2,746,388 as of 2020, making it the third-largest city in the U.S. after New York City and Los Angeles. It is the seat of Cook County and the center of the Chicago metropolitan area, known as “Chicagoland”. The data include occurrence time stamp, longitude, latitude, crime types, description of crime, etc.. The catalog is publicly available at [42]. The data set consists of 29 types of crimes. We focus only on the burglary crime subset, comprising 25,201 events occurring in Chicago within a rectangular area defined by longitudes –87.91° to –87.53° and latitudes 41.65° to 42.02°. The longitudes and latitudes are transferred to plane coordinates measured in kilometers using the locally scaled equirectangular projection. This transformation is based on a local linearization of the earth’s surface around the study area. Following this transformation, the spatial dimensions were shifted to start from zero by subtracting the minimum transformed value along each axis resulting in spatial region defined in kilometers.

### Fitting the burglary data

**Fig 6:**
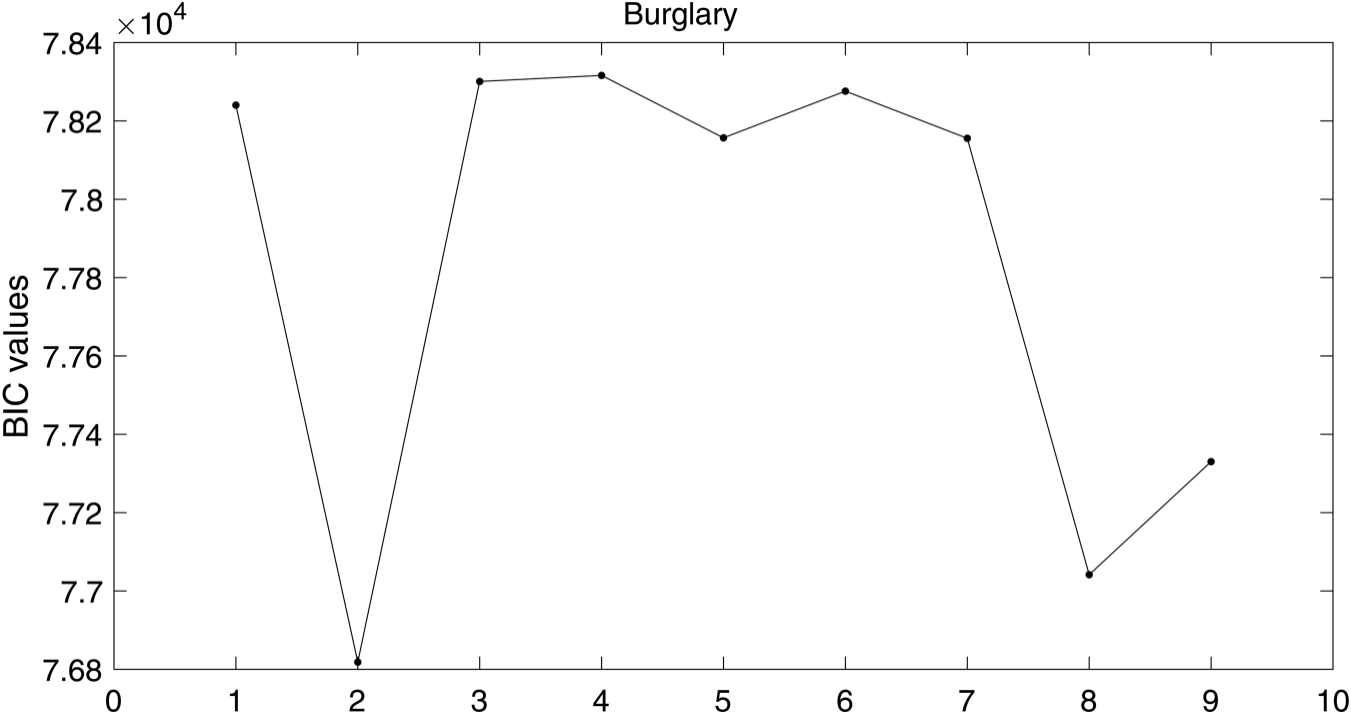
BIC-based model selection using the proposed estimation method.

**Fig 7:**
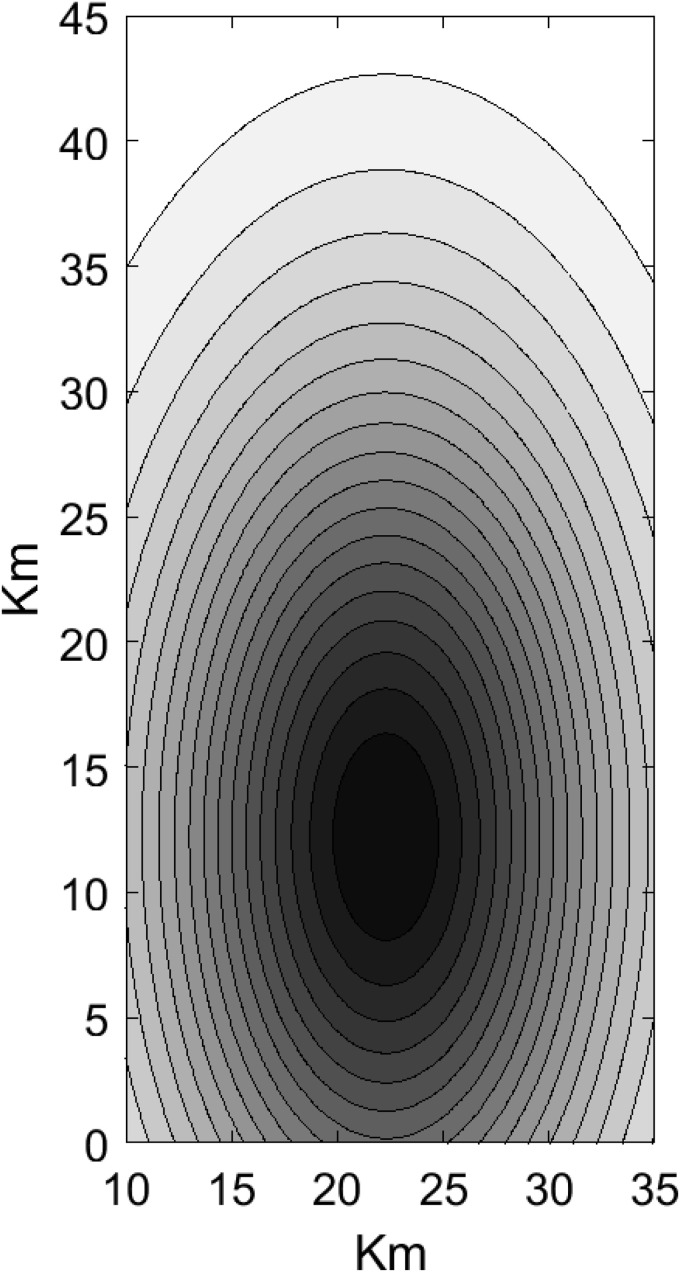
Space marginal μ(x,y) estimated using proposed method.

**Fig 8:**
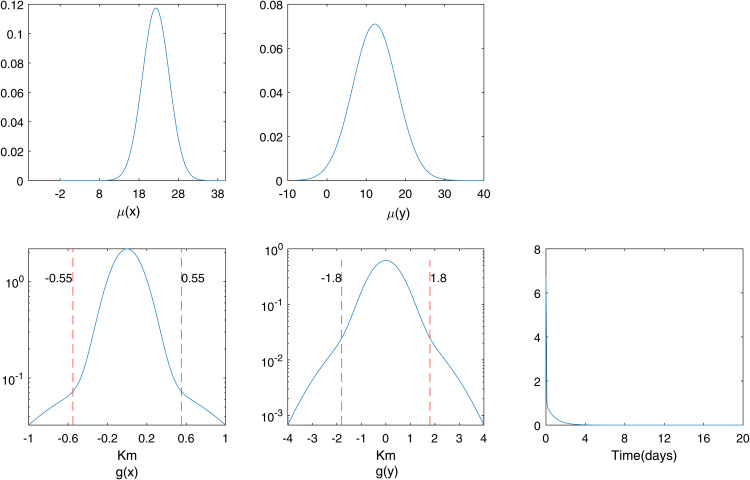
Background rate marginals, μ(x) and μ(y), as well as the triggering effect marginals, *g*(*x*) and *g*(*y*), estimated using proposed method.

**Fig 9:**
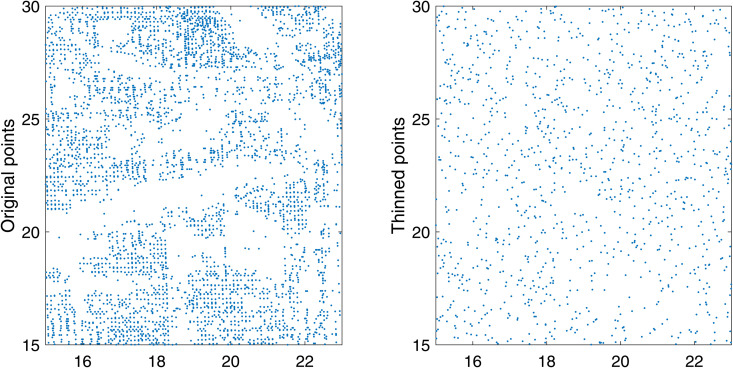
The original location of the selected burglaries and the generated location with super-thinning.

Following the specification of the background and triggering components, we proceed with model fitting using the spatiotemporal burglary data. Our goal is to estimate the space-time background function, which represents the occurrence rate of spontaneous burglaries. The space-time background function μ(x,y,t), which varies across the observation region, is estimated using the multi-dimensional Gaussian-type exponent approximation method introduced in the previous section. The estimation results are presented in Figs 6–9. Using the BIC-based model selection method in Fig 6, we select the model with the smallest BIC value which corresponds to (*p*,*q*) = (0,1).

Modeling the burglary data using the proposed model, we first examine the triggering effect, which characterizes the spatiotemporal clustering of burglaries. From the second row of Fig 8, we can see a plot of the marginal density estimates of the triggering effects. The clustering features are vividly observed, as these spatial marginal densities approximate the distribution with minimal variance. Observation from Fig 8 indicates that initial burglaries may trigger other burglaries within a close distance and short time frame. This is because burglars may target nearby houses after successfully executing their initial plans. The burglaries occur within neighborhoods of approximately 0.5 km × 1.8 km and within the first few days (1–3 days). The burglaries are more concentrated within localized areas over specific time periods, but diminish as distance and time increase.

Fig 7 and the first row of Fig 8 shows the marginal densities of the estimated background process, which represents the rate of occurrence of spontaneous events, untriggered burglaries. The orientation of Fig 7 suggests that there is no correlation between the spatial components; hence, it appears aligned with the axes rather than slanted, indicating that the spatial components are independent.

**Fig 10:**
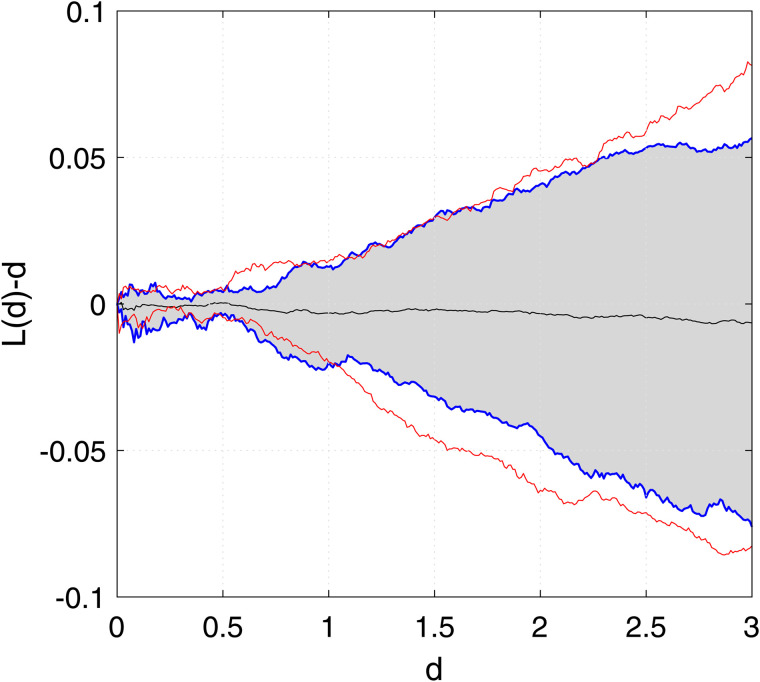
Super-thinning residuals for the Chicago burglary data set. The lower red curve represents the 5% bounds of the estimated centered *L*-function, *L*(*d*)–*d*, for a homogeneous Poisson process, while the upper red curve corresponds to its 95% bounds. Similarly, the lower blue curve denotes the 5% bounds of the estimated centered *L*-function for the thinned residuals, and the upper blue curve indicates its 95% bounds. The middle black line represents the empirical mean of the estimated centered *L*-function for the thinned residuals, while the gray-shaded region illustrates the confidence interval of the estimated centered *L*-function for the thinned residuals.

We evaluate the goodness-of-fit of the proposed multi-dimensional Gaussian-type exponent approximation for the self-exciting point process within a selected region of size [15,23]× [15,30]. The implementation of the super-thinning method is illustrated in Fig 9, where we observe that the super-thinning process exhibits behavior similar to a Poisson process. Specifically, the first column of Fig 9 shows the selected spatial region used for model evaluation, while the second column illustrates the points that remain after applying the super-thinning procedure. In Fig 10, we present the computed values of the centered *L*-function, expressed as *L*(*d*)–*d*, along with the 95% confidence bounds for a homogeneous Poisson process within the designated area. The results indicate that the estimated centered *L*-function values fall within the 95% confidence limits as shown in Fig 10. This finding indicates that the proposed multi-dimensional Gaussian-type exponent approximation method for the self-exciting point process can effectively model burglary crimes.

## 6 Conclusion

We introduce a fast algorithm that uses a multi-dimensional Gaussian-type exponent approximation to estimate the background intensity and triggering effect of a space-time self-exciting point process model based on space-time datasets. This method offers significant advantages over other estimation methods, particularly for space-time self-exciting models, in terms of flexibility and computational efficiency. The method employs the BIC criterion to select the appropriate model order, which can be either single component or multi-component. The proposed multi-dimensional Gaussian-type exponent approximation method demonstrates strong estimation performance in both simulation studies and the burglary data application. Although some parameter estimates exhibit slight bias, our model, despite not incorporating the ρ parameter from the Gaussian function, still performs well. As the data size increases, the method improves and obtains close approximations.

Furthermore, we demonstrate how space-time self-exciting point processes can be applied to model burglary crime. The proposed model captured the clustering behavior of the burglary data. This clustering reflects the underlying mechanisms of event triggering and propagation, in which prior burglary incidents increase the short-term risk of subsequent crimes in nearby locations. This behavior illustrates how information cascades about criminal activity spread both spatially and temporally, influencing offender decision-making. The proposed method is not limited to burglary; it is also applicable to other areas involving counting data characterized by self-excitation or event cascades, such as earthquakes (aftershock sequences), financial transactions (volatility clustering), and information diffusion processes. In these settings, efficiently capturing triggering effects induced by past events is essential for both interpretation and prediction. A current limitation of the model is its assumption of independence among spatial coordinates, as it does not include a spatial covariance structure. Although spatial dependence is illustrated in the simulation study through a correlation parameter ρ, this dependence is not modeled in the empirical estimation.

For future work, we aim to explore the use of separable temporal components for the background and triggering intensity functions, as well as investigate other properties of the model through second-order residual analysis. Future work will investigate the model’s covariance using the Gaussian mixture model (GMM). The next step of the study is to introduce covariance differentials.
